# Auxin secretion by *Bacillus amyloliquefaciens* FZB42 both stimulates root exudation and limits phosphorus uptake in *Triticum aestivum*

**DOI:** 10.1186/1471-2229-14-51

**Published:** 2014-02-21

**Authors:** Peter J Talboys, Darren W Owen, John R Healey, Paul JA Withers, David L Jones

**Affiliations:** 1 School of Environment, Natural Resources and Geography, College of Natural Sciences, Bangor University, Bangor, Gwynedd LL57 2DG, UK

**Keywords:** *Bacillus amyloliquefaciens* FZB42, Seed treatment, Wheat, Auxin, Phosphate, Root, Exudation

## Abstract

**Background:**

The use of auxin-producing rhizosphere bacteria as agricultural products promises increased root production and therefore greater phosphate (Pi) uptake. Whilst such bacteria promote root production in vitro, the nature of the bacteria-plant interaction in live soil, particularly concerning any effects on nutrient uptake, are not known. This study uses *Bacillus amyloliquefaciens* FZB42, an auxin-producing rhizobacterium, as a dressing on *Triticum aestivum* seeds. It then examines the effects on root production, Pi uptake, Pi-related gene expression and organic carbon (C) exudation.

**Results:**

Seed treatment with *B. amyloliquefaciens* FZB42 increased root production at low environmental Pi concentrations, but significantly repressed root Pi uptake. This coincided with an auxin-mediated reduction in expression of the Pi transporters Ta*PHT1.8* and Ta*PHT1.10*. Applied exogenous auxin also triggered an increase in root C exudation. At high external Pi concentrations, root production was promoted by *B. amyloliquefaciens* FZB42*,* but Pi uptake was unaffected.

**Conclusions:**

We conclude that, alongside promoting root production, auxin biosynthesis by *B. amyloliquefaciens* FZB42 both re-models Pi transporter expression and elevates organic C exudation. This shows the potential importance of rhizobacterial-derived auxin following colonisation of root surfaces, and the nature of this bacteria-plant interaction in soil.

## Background

Microbial formulations are used as additives in agriculture promising stimulation of root production, thus enhanced uptake of water and nutrients, resistance to pathogens and increased resilience to environmental stresses such as drought, salinity and heavy metal contamination [1-10]. Soil microbes can also play a more direct role in plant nutrient acquisition, especially for those nutrients that are inherently less available in soils, such as phosphorus (P) [11]. Microbial metabolism is dependent on a source of labile carbon (C) and the rhizosphere is far richer in microorganisms than the surrounding bulk soil due to the substantial exudation of C by plant roots. Bacteria colonise only a small proportion of the root surface, largely the junctions between epidermal cells and the regions surrounding emerging lateral roots where C is secreted [12,13]. A number of these rhizobacteria species increase root production through effects on plant hormonal signalling processes: either by production of hormones in the bacteria themselves [14-16] or by perturbation of endogenous concentrations [17] or transport [18] within the plant. However, evidence to support positive yield benefits from the use of individual strains, or commercial mixtures, of rhizobacteria in field soils is mixed, suggesting an incomplete understanding of the mechanisms and interactions involved. This study focuses specifically on the auxin-producing bacterium *Bacillus amyloliquefaciens* FZB42, and the nature of the resulting plant-microbe interactions involved in plant P uptake.

Auxin is a plant hormone which regulates a large number of root biological processes including the regulation of cell division and differentiation in processes as diverse as root hair production, meristem maintenance, root gravitrophism and lateral root production. A large proportion of rhizosphere bacteria synthesise auxin [19-21], and it is proposed that this is responsible for the promotion of root growth by plant-associated *Azospirillum*, *Bacillus*, *Pseudomonas* and *Rhizobium* species [19]. This auxin production is hypothesised to be a component of a bacterial colonisation mechanism whereby the auxin-induced stimulation of root growth and branching leads to an increase in the area available for bacterial colonisation and so increased C supply [19]. The process of auxin production has been shown to be similar in bacteria and plants [18], and is often sensitive to environmental tryptophan (a precursor of auxin) levels [14,16,22-24].

One of the major objectives of the increase in root production stimulated by microbial inoculation is the increased acquisition of phosphorus (P). The concentration of freely available inorganic P (Pi) in soil solution is typically very low, due to its propensity to bind strongly to soil surfaces or form insoluble complexes with cations [25]. This means that Pi availability is often a limiting factor in plant growth and development, and so increasing a plant’s ability to forage for Pi is desirable for crop production. A number of physiological factors determine Pi uptake efficiency in cereals including: lateral root branching and elongation [26]; root hair density [27]; exudation of organic acid anions and phosphatases into the rhizosphere [28]; and formation of symbioses with mycorrhizal fungi [29]. The technical difficulties involved in using bioengineering to exploit these traits [30], alongside some nations’ reluctance to embrace such technologies, has contributed to the use of bacterial and fungal inoculants to improve crop Pi uptake ability and therefore increase yields [31].

*B. amyloliquefaciens* FZB42 is a plant growth-promoting bacterium that has been demonstrated to promote the growth of roots it has colonised [32]. This strain has been shown to produce large quantities of auxin, with increases in production following the addition of tryptophan to the culture media [14]. This study aims to assess the nature of the biological interaction between *B. amyloliquefaciens* FZB42 and the *Triticum aestivum* root system: with the hypothesis that any effects of *B. amyloliquefaciens* FZB42 colonisation on root Pi uptake rates are dependent upon the soil Pi concentration. As the primary mode of *B. amyloliquefaciens* FZB42-root interaction observed so far is auxin production [14], this interaction was investigated further by analysing the effects of exogenous auxin application upon both root Pi-related gene expression, and root exudation of organic C.

## Results

### *Bacillus amyloliquefaciens* FZB42 effect on root production

**Figure 1:**
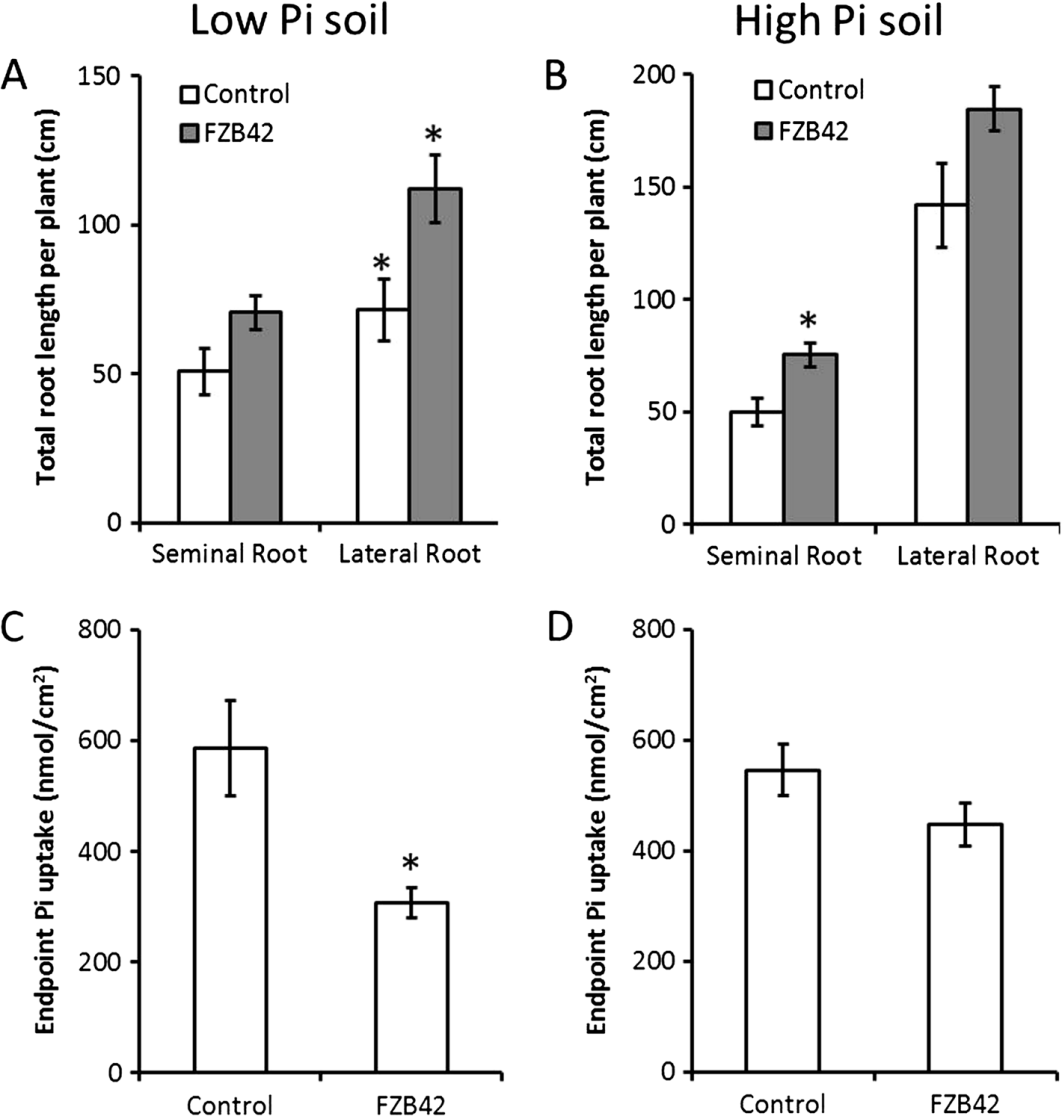
**Seed dressing with *B. amyloliquefaciens *FZB42 stimulates increased root production but reduced Pi uptake per root surface area in low Pi soils but not high Pi soils. A**-**B** Total length of each root class per plant for three-week-old plants seed dressed at sowing with either *B. amyloliquefaciens* FZB42 or LB media as the untreated control and grown in either low Pi (A) or high Pi soil (B). **C**-**D** The total plant Pi content of the treatments in A and B, expressed as total Pi acquired after three weeks per root surface area. For A - D, n > 23 for each treatment, and *marks values significantly different from the untreated control values using Student’s t-test (p < 0.05). Error bars are standard errors of the mean (SEM).

In the experimental conditions described above, the application of *B. amyloliquefaciens* FZB42 as a seed dressing stimulated *T. aestivum* root production in live soil of both low and high Pi concentration (Figure 1A,B). Treatment resulted in significant increases in the length of the seminal root (by 39.1%) and first order lateral root (by 51.0%) per plant under *B. amyloliquefaciens* FZB42 treatment in low Pi soil (Figure 1A), and length of the seminal root per plant (by 50.9%) in high Pi soil (Figure 1B).

### *Bacillus amyloliquefaciens* FZB42 impacts Pi uptake from low Pi environments

The average total Pi uptake per plant after three weeks growth was not significantly different in the *B. amyloliquefaciens* FZB42 treatment from the un-inoculated controls (Additional file 1: Figure S1A, B) in both low- and high-Pi soils. When expressed on a per unit root surface area basis, however, *B. amyloliquefaciens* FZB42 treated root systems grown in low Pi soils were significantly less effective at acquiring Pi than un-inoculated controls (Figure 1C). There was, however, no significant difference in endpoint Pi uptake per unit root surface area between un-inoculated controls and *B. amyloliquefaciens* FZB42 treatments in high Pi soils (Figure 1D).

**Figure 2:**
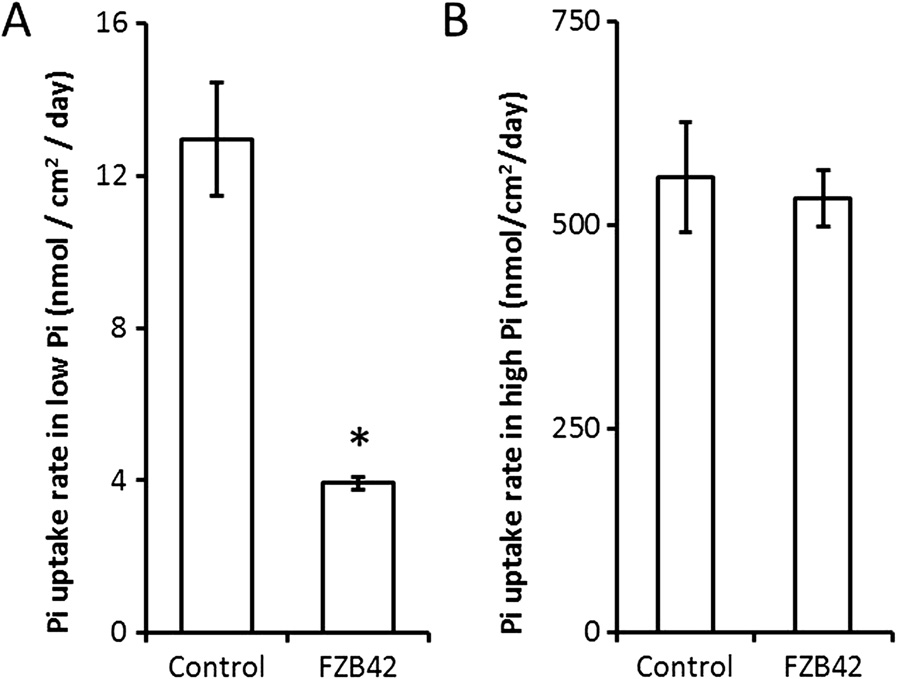
***B. amyloliquefaciens *FZB42 represses root Pi uptake in only low Pi environments. A** and **B** Uptake rates from one-week-old *T. aestivum* plants that had been seed treated with 2 µl of either LB media or *B. amyloliquefaciens* FZB42 culture, where the soil was watered to holding capacity with either A 20 µM PO_4_^3-^ or B 2 mM PO_4_^3-^. Both solutions were supplemented with ^33^P whose presence was used to monitor Pi uptake. n = 5 for each treatment, and * marks values significantly different from the untreated control value using Student’s t-test (p < 0.05). Error bars are SEM.

One week old, soil-grown, *T. aestivum* root systems were grown in soil with either a high or low exogenous Pi supply: it was found that *B. amyloliquefaciens* FZB42 seed-treatment resulted in a reduced Pi uptake rate under low external Pi conditions compared with the un-inoculated controls (Figure 2A). Plants had a significantly lower rate of Pi uptake per root surface area than the controls (Figure 2A), with the average Pi uptake being 152.4 nmol plant^-1^ (control) and 50.8 nmol plant^-1^ (*B. amyloliquefaciens* FZB42). However, under a higher exogenous Pi supply (irrigated with a 2 mM Pi solution) there was no significant difference between the treatments (Figure 2B), with the larger root system of the *B. amyloliquefaciens* FZB42 treated plants acquiring an average of 555.6 nmol plant^-1^ Pi compared with the 458.7 nmol plant^-1^ Pi of the controls.

### Impact of *Bacillus amyloliquefaciens* FZB42 on P transporter expression

**Figure 3:**
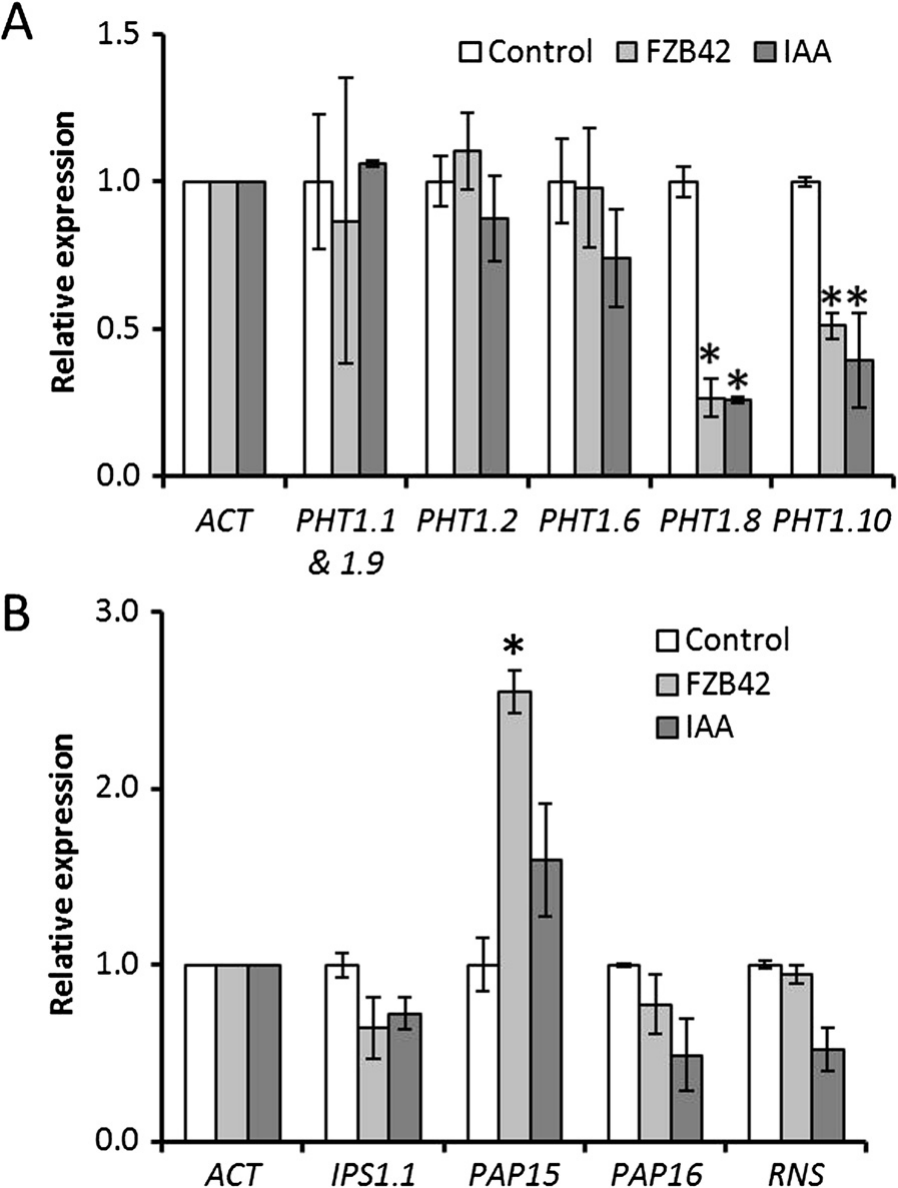
***B. amyloliquefaciens *FZB42 treatment effects on expression of root Pi transporters, and genes involved in internal Pi metabolism. A** Relative expression of *T. aestivum PHT1* genes, in roots, standardised to *actin* (*ACT*) controls. The experimental treatments were: inoculation with *B. amyloliquefaciens* FZB42*,* treatment with 1 µM IAA and untreated controls. **B** Relative expression of *T. aestivum* Pi metabolism genes in roots, standardised to *actin* (*ACT*) controls with the same treatments as in A. For A and B n = 3 for each treatment, error bars are SEM, and * marks values significantly different from the untreated control value using Student’s t-test (p < 0.05).

The relative expression of genes encoding individual cellular Pi transporters was also assayed. Of the six Pi transporters tested, a significant reduction was found in the relative expression of Ta*PHT1.8* and Ta*PHT1.10*, in both the *B. amyloliquefaciens* FZB42 and IAA treatments relative to the control (Figure 3A). Of the five Pi-associated genes tested, only *PAP15* showed any significant difference in expression under *B. amyloliquefaciens* FZB42 treatment relative to the control, with its expression increasing 154.7% over two fold over untreated controls (Figure 3B). The expression of none of these genes was significantly affected by the IAA treatment (Figure 3B).

### Impact of IAA on root exudation

**Figure 4:**
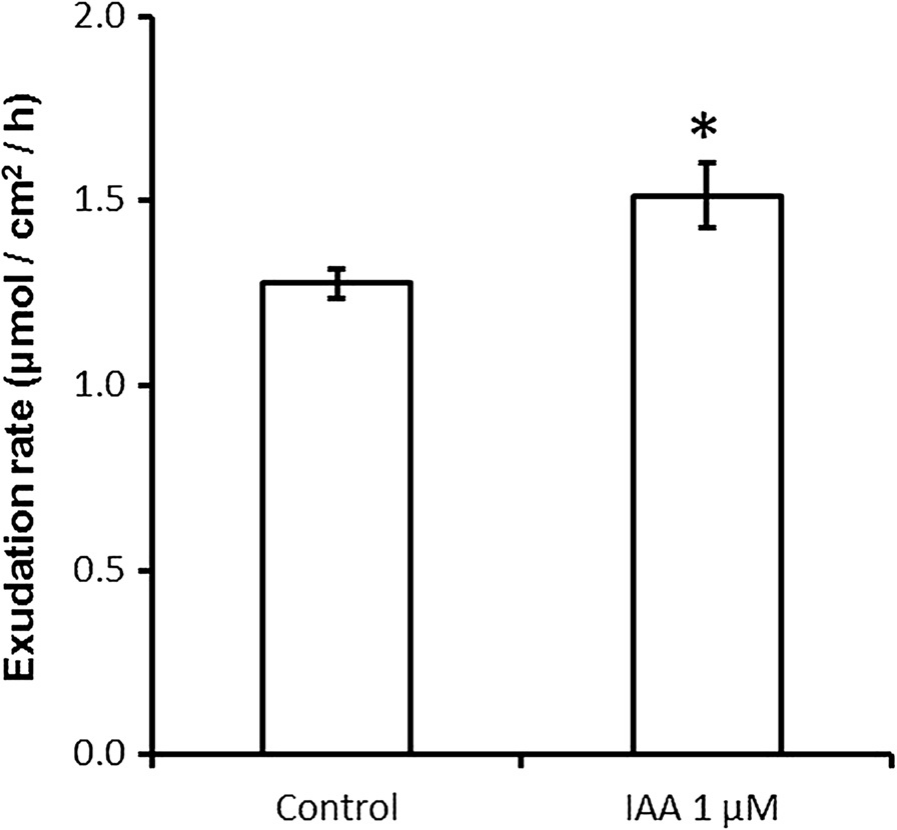
**Exogenous IAA application results in greater C exudation rates from *T. aestivum *roots**. The release rate of ^14^C tagged 3-O-M-glucose from one-week-old *T. aestivum* root systems submerged either in 1 µM IAA or untreated deionised water controls. n = 5 for each treatment, error bars are SEM, and * marks values significantly different from the untreated control value using Student’s t-test (p < 0.05).

The exogenous application of IAA to 1-week-old *T. aestivum* root systems was assessed using 3-O-M-glucose as a marker of sugar exudation. The application of 1 mM IAA produced a significant increase in 3-O-M-glucose release when expressed as a function of root system surface area (Figure 4).

## Discussion

### Auxin mediated bio-stimulation of *T. aestivum* root system growth comes at a cost in Pi uptake rate

This study presents new experimental data demonstrating the complex nature of plant-microbial interactions in the rhizosphere. *B. amyloliquefaciens* FZB42, like a large proportion of rhizosphere microbes, secretes auxin as a component of its interaction with the plant [14]. The results of the present study demonstrate that exogenous auxin application to the *T. aestivum* root system, whilst capable of stimulating an increase in root production, also reduced root Pi uptake rate per unit root surface area from low Pi soils (Figure 1C, Figure 2A).

Root Pi uptake is mediated by Phosphate Transporter (PHT) proteins which are phosphate : H^+^ symporters that use electrochemical gradients to drive Pi and proton symport into the plant [33]. In *T. aestivum* the Ta*PHT* gene family is proposed to encode a group of closely related high affinity phosphate transporter proteins whose expression in root tissue has been shown to be increased (Ta*PHT1.1 & 1.9, 1.2, 1.8, 1.10*) or unchanged (Ta*PHT1.6*) in response to decreased root Pi supply [34,35]. Ta*PHT1.1 & 1.9, 1.2* and *1.10* expression has conversely been shown to be reduced in response to low P supply under field conditions, but this has been attributed to the effects of high levels of mycorrhizal colonisation [34]. Given that Ta*PHT1.8* and Ta*PHT1.10* have previously been identified as having significantly up-regulated expression in roots growing in low Pi environments [34], the lower Pi uptake rates displayed by *B. amyloliquefaciens* FZB42 treated plants in low Pi conditions in the present study (Figure 1C, and Figure 2A) could be explained by the *B. amyloliquefaciens* FZB42 treatment reducing Ta*PHT1.8* and Ta*PHT1.10* expression (Figure 3A). The *B. amyloliquefaciens* FZB42 treatment effect upon Ta*PHT1.8* and Ta*PHT1.10* expression was also mimicked by the exogenous IAA application, which strongly implies that this is at least in part an auxin mediated process, and so an intrinsic component of the *B. amyloliquefaciens* FZB42-root interaction (Figure 3A). We therefore propose that *B. amyloliquefaciens* FZB42 auxin production lowered root Ta*PHT1.8* and Ta*PHT1.10* expression, directly resulting in the depressed plant Pi uptake levels observed in the pot experiments (Figure 1C). It is possible that this effect enables auxin producing rhizobacteria to better compete for localised P in low P soils: auxin application has previously been shown to perturb the expression pattern of *PHT* genes in *Arabidopsis*[36], and microbial re-modelling of *PHT* expression has also been demonstrated using arbuscular mycorrhizal fungi [37,38]. Our results have implications for the use of auxins in future biotechnological applications. Specifically, the use of auxins, or auxin-producing micro-organisms, to stimulate root production should be carefully mapped to environmental P conditions to ensure optimal Pi uptake, plant growth and yield.

### Internal P-mobilisation gene expression

Treatment with *B. amyloliquefaciens* FZB42 resulted in the up-regulation of *PAP15* expression, whereas the levels of *PAP16*, *IPS1.1* and *RNS* remained unchanged (Figure 3B). This increase in *PAP15* expression is potentially a homeostatic adjustment by the plant to recycle more P, counteracting the reduced Pi uptake caused by the auxin released by *B. amyloliquefaciens* FZB42 treatment. The lack of an *IPS1.1* expression response in the *B. amyloliquefaciens* FZB42 and IAA treated roots indicates that the effects of auxin upon plant Pi gene expression is nuanced rather than a wholesale shift in perceived Pi status.

### *Bacillus amyloliquefaciens* FZB42 seed treatment in higher Pi status soils

In contrast to low Pi soil, there was no significant negative effect of *B. amyloliquefaciens* FZB42 treatment on Pi uptake in soil with a high Pi concentration (Figure 1D, and Figure 2B). This could be due to the unchanged expression levels of Ta*PHT1.2,* Ta*PHT1.1 & 1.9* (Figure 3A) which are still expressed in high Pi environments, or the presence of Ta*PHT1.6* which is expressed in roots independently of environmental Pi concentration [24]. Ta*PHT1.10* expression was found to be significantly reduced under *B. amyloliquefaciens* FZB42 treatment (Figure 3A), but is also highly homologous to Ta*PHT1.2* and Ta*PHT1.1 & 1.9*[35]. This implies that, despite their proposed functional similarity, their expression is controlled by different factors.

*B. amyloliquefaciens* FZB42 treatment did, however, induce a beneficial effect on plant root production in high Pi soils (Figure 1B) meaning that, under these conditions, the plants were capable of realising the many benefits of a large root system, such as increased uptake of water and nutrients, without the pitfall of suppressed Pi uptake per root surface area. It is interesting to note that the size of this increase was far smaller than that in low Pi soils, probably due to this effect being partly swamped by the increases in root elongation and root branching that are normally observed after elevating environmental Pi [39]. Therefore the use of *B. amyloliquefaciens* FZB42 treatment within high Pi soils, or alongside conventional Pi fertilisation, is potentially beneficial to agricultural systems: the auxin produced providing enhanced root production with none of the negative effects upon Pi uptake observed in low Pi environments in this study (Figure 2A,B).

### Stimulation of root organic C exudation provides an insight into rhizobacteria auxin production

As the rate of microbial metabolism in most agricultural soils is limited by the availability of labile C compounds [40], the rate of organic C exudation from root systems has a great influence on the microbial community. This results in an elevated bacterial population in the rhizosphere compared with the bulk soil [41]. It has been estimated that as many as 80% of bacterial species populating the rhizosphere produce auxin, where they are hypothesised to be the beneficiaries of an increase in colonisable root area through auxin stimulated root production [20,21]. The results of the present study shown in Figure 4 indicate that the resulting elevated concentrations of auxin in the rhizosphere can stimulate increased net exudation of sugars from plant roots. As the exudation of sugars from cereal roots is largely a passive process [42], it would imply that auxin has either (1) down-regulated the plasma membrane H^+^-ATPase driven hexose H^+^-co-transport which recaptures sugars lost from the root, or (2) increased membrane permeability facilitating faster efflux. The latter hypothesis is supported by studies in *T. aestivum* callus cultures and maize membrane vesicles showing higher membrane permeability and ion leakage from cells treated with IAA and minimal direct effect on H^+^-ATPase functioning [43,44]. That this increased sugar loss was measurable after only one hour in the present study implies that it is likely to be a direct plant root response to auxin rather than an indirect effect. This is a previously un-described effect of bacterial auxin production, and a priority for future research is to assess the response relationship to the dose of auxin applied, with reference to known rates of production from rhizosphere bacterial communities.

The results of the present study shown in Figure 4 also have wider implications for the mechanisms by which the rate of organic molecule exudation by plant roots is controlled. Organic acids have been shown to be exuded by the roots of many species in response to Pi deficiency, displacing Pi from inorganic precipitates found in the soil [45]. The root tip and root hairs have been shown to be the site of the bulk of this exudation [46,47], and the root tip is also the location of significant auxin response maxima, the pattern of which is tightly controlled [48]. Alterations in the auxin fluxes within the root tip are therefore a potential mechanism for controlling C exudation by roots. This process could synergistically promote root uptake of Pi in low Pi environments, with greater concentrations of exuded organic acid anions both: chemically displacing Pi from insoluble soil complexes [49], and increasing the labile C source available to rhizosphere microbes which may accelerate biological mobilisation of soil organic P [50].

### Longevity of *Bacillus amyloliquefaciens* FZB42 treatment effects

In the direct ^33^Pi uptake experiments of the present study the *B. amyloliquefaciens* FZB42 treatment caused a proportionally greater reduction in Pi uptake in the low Pi status soil after one week (Figure 2A) than was observed at the end point of the three week pot trial (Figure 1C). It is possible, therefore, that after the first week of growth, the *B. amyloliquefaciens* FZB42 has been increasingly outcompeted at the root surface by endogenous soil microbes [11,51]. This assumes that *B. amyloliquefaciens* FZB42 colonisation of the seed coat has enabled root surface colonisation as seen in *Bacillus* treatment of *Zea mays* seeds [52]. However, even if this was the case, the greater size of root system established during the crucial first three weeks of root development in *T. aestivum* is likely to persist. This would therefore provide potential longer-term benefits for increased crop yield.

## Conclusions

One consequence of the demand for increased agricultural food production is the growth in the use of bio-stimulatory products that are marketed on the basis of increasing crop nutrient uptake for a much lower cost than that of extra nutrient application in fertiliser. *B. amyloliquefaciens* FZB42, like many rhizosphere microbes, produces auxin which is likely to be a major component of its interaction with the root system of crop plants such as *T. aestivum*. The study demonstrates that *B. amyloliquefaciens* FZB42 auxin production can drive a reduction in the expression of the high-affinity Pi transporters Ta*PHT1.8* and Ta*PHT1.10*, alongside a reduction of the root’s capacity to take up Pi from soils with a low Pi concentration. At the same time *B. amyloliquefaciens* FZB42 stimulates a greater rate of early growth of both seminal and lateral roots in *T. aestivum*. The results presented here provide new insight into the complex biological interactions between the *T. aestivum* root system and auxin producing bacteria in the rhizosphere, and the potential trade-off of these different effects for crop P nutrition. The reduced root Pi uptake caused by the addition of *B. amyloliquefaciens* FZB42*,* and its interaction with soil Pi concentration, highlights the importance of acquiring a detailed understanding of the interaction of any new microbial strain with crop root systems across a range of environmental conditions, before their widespread use in agriculture is advocated. This should reduce the risk of unanticipated adverse effects, identify how the efficacy of different products varies with environmental conditions and provide farmers with the evidence to make an informed cost-benefit analysis of the value of such products compared with alternatives.

## Methods

### Pot trial growth conditions

Cultures of *Bacillus amyloliquefaciens FZB42* (Bacillus Genetic Stock Center, Columbus, OH, USA) were grown in Luria Broth (LB) medium in a rotary shaker until they reached an Optical Density at 600 nm (OD_600_) of 3.0 (2.1 × 10^10^ CFU ml^-1^). *Triticum aestivum* seeds of the commercial variety PARAGON (RAGT Seeds Ltd, Saffron Walden, UK) were then coated with 2 µl of this solution or untreated LB medium (untreated controls). This seed coating reflects the cell density provided by the commercial *B. amyloliquefaciens* FZB42 containing product Biomex Starter (6.25 × 10^10^ CFU ml^-1^; OMEX Agriculture Ltd). Cell density was assessed by culturing serial dilutions of the stock culture on LB agar plates. Excess water from the treatments was allowed to evaporate. Three seeds were then planted in each 8 cm diameter pots filled with 300 g of one of two soils. The soils used were both sandy loams, with plant-available (Olsen P) concentrations of 14.5 mg kg^-1^ (Abergwyngregyn, UK; Table 1, Figure 1A,C) and 36.5 mg kg^-1^ (Thonock, UK; Table 1, Figure 1B, D). The pots were then placed in a climate-controlled greenhouse maintained at 20°C and supplemented with artificial lighting (light intensity = 260 µmol m^-2^ s^-1^ PAR) with a minimum photoperiod of 16 h. The number of seedlings was thinned to one per pot at emergence, and the soil in the pots was maintained at 80% of its water holding capacity by watering thrice weekly. Soil water holding capacity was measured gravimetrically [53]. To ensure that P was the only limiting macronutrient, the equivalent of 60 kg ha^-1^ N (as 1 M NH_4_NO_3_ solution) and 60 kg ha^-1^ K (as 1 M KCl solution) were applied to each pot at seedling emergence. Micronutrients were controlled by the weekly application of 10 ml of a solution containing: 5 mM Ca; 3.87 mM Fe; 3.87 µM Na; 765 µM Zn; 2 mM SO_4_; 320 nM Cu; 46.3 nM BO_3_; 500 µM Mo; 9.1 nM Mn; 18 µM Cl; 38.7 µM EDTA.

**Table 1:** Characteristics of the soils used in the pot experiments

**Characteristic**	**Low P soil**	**High P soil**
Textural class	Loamy sand	Loamy sand
pH	5.9	7.0
Available P (Olsen) (mg kg^-1^)	14.5	36.5
Available K (mg kg^-1^)	163.9	68.0
Available Mg (mg kg^-1^)	83.6	114.8

This table shows the textural class, pH and available P, K and Mg of the soils used in the pot experiments. These values were measured by NRM Ltd., UK and are expressed as mg per kg dry soil.

### Pot trial measurements

At 21 days after sowing the plants were harvested. The roots were washed thoroughly in distilled water, floated out on water in transparent plastic trays, and scanned using a flatbed scanner (Perfection 4990 Photo; Epson Electronics America Inc., San Jose, CA, USA). The resulting image was processed using WinRhizo® software (Regent Instruments Inc., Canada) to determine the length of seminal and lateral roots per plant, and the resulting root system surface area. The boundary conditions used by the software were that roots ≥0.350 mm in diameter were classified as seminal roots, and < 0.350 mm were classed as lateral roots (J. Heppell, Personal communication). The whole plants were then dried at 85°C overnight, weighed, and dry-ashed (550°C, 16 h). The residue was dissolved in 0.5 M HCl and then their P content determined using the ascorbate/molybdate blue method of Murphy & Riley, [54].

### Direct Pi uptake measurements

A separate set of plants were grown as described above, but one week after sowing the soil was watered to holding capacity with either 20 µM or 2 mM K_2_HPO_4_ solution combined with 1 kBq ml^-1^^33^P (American Radiolabeled Chemicals Inc., St Louis, MO, USA). After 24 h of incubation at 20°C, the plants were harvested, washed and the root surface areas were measured using WinRhizo®, as described above. The whole plants were dried at 85°C overnight and then dry-ashed (550°C, 16 h). The residue was dissolved in 0.5 M HCl and the ^33^P content of the resulting solution was quantified using a Wallac 1404 scintillation counter (Wallac EG&G, Milton Keynes, UK). The quantity of ^33^P found in the plant, divided by the quantity of ^33^P added to the soil multiplied by the total P concentration of the solution added to the soil was then used to calculate the amount of applied Pi acquired by the root system.

### Organic C exudation assay

Plants were grown as for the pot trial, but one week after sowing they were harvested intact and the root systems were gently washed to remove the soil, before being submerged in 1 mM 3-O-methyl-D-glucose solution (3-O-M-glucose). 3-O-M-glucose is a non-metabolisable analogue of glucose that is routinely used to quantify the rate of sugar influx and efflux across plant and animal membranes [55-58]. The 3-O-M glucose solution was supplemented with 1 kBq ml^-1^ of ^14^C-3-O-M-glucose (American Radiolabeled Chemicals Inc., USA). After 24 h, the root bathing solution was replaced to remove any remaining ^14^C-labelled 3-O-M-glucose. The root bathing solution was then replaced with either deionised water, or 1 µM of the auxin indole acetic acid (IAA; Sigma Aldrich, Poole, UK) and the roots incubated for 1 h to determine the net rate of 3-O-M-glucose efflux. Such alterations in monosaccharide efflux are generally rapid processes [56-58], as are many auxin responses [59], therefore one hour was deemed sufficient to assess any effect. After the plants were removed, the ^14^C content of the resulting root bathing solution was quantified using a Wallac 1404 scintillation counter (Wallac EG&G, Milton Keynes, UK). The root system surface areas of each plant were then determined using WinRhizo® as described above.

### Quantitative RT-PCR analysis

*T. aestivum* seeds were pre-germinated by submerging in aerated deionised water overnight. The seeds were then placed in Petri-dishes containing filter paper soaked in a modified Hoagland’s solution containing: 5.5 mM K; 110 mM NO_3_; 1 mM NH_4_; 5 mM Ca; 3.87 mM Fe; 3.87 µM Na^+^; 765 µM Zn; 2 mM SO_4_; 320 nM Cu; 46.3 nM BO_3_; 500 µM Mo; 9.1 nM Mn; 18 µM Cl; 38.7 µM EDTA and 5 µM PO_4_. Seeds coated with *B. amyloliquefaciens* FZB42 (and negative controls) were prepared as for the pot trial, whilst for the IAA treatment, 1 µM of IAA was added to the low Pi medium. The plants were then incubated at 20°C for 40 h. The roots were then removed, with all seminal roots from five plants forming one replicate (three such replicates were then used per treatment), and the total RNA was extracted using a GeneMATRIX RNA/miRNA purification kit (Roboklon, Berlin, Germany). A dART RT (Roboklon, Germany) kit was then used to construct cDNA from this RNA extract using oligo d(T) primers. Quantitative RT-PCR was performed using an Applied Biosystems thermocycler (Life Technologies Ltd, Paisley, UK) with a SYBR Green qPCR mix (Roboklon, Germany), and normalised to actin [GenBank: AB181991] controls performed using primer pairs published by Teng *et al*. [34]. The genes assayed were: the putative Pi transporter encoding genes Ta*PHT1.1 & 1.9* [GenBank: AJ344241], Ta*PHT1.2* [GenBank: AJ344240], Ta*PHT1.6* [No published sequence], Ta*PHT1.8* [GenBank: AJ830009] and Ta*PHT1.10* [No published sequence]; *IPS1.1* [Genbank: EU753150.1] is a molecular marker for plant P starvation whose expression correlates with plant P status [34,60,61]; *PAP15* [GenBank: CJ554973] and *PAP16* [No published sequence] genes encode putative purple acid phosphatases [34] which hydrolyse phosphate esters and anhydrides to help both recycle P within the plant, and mobilise organic P in soil [62]; and *RNS* [GenBank: AY517470] encoding a S-like ribonuclease which remobilises P from RNA [63].

### Statistical analysis

The statistical test for significance used throughout was student’s t-test, which was carried out using MS Excel (Microsoft Corp., Redmond, WA, USA). Only values where p < 0.05 were treated as significantly different.

## Abbreviations

P: Phosphorus; Pi: Phosphate; C: Carbon.

## Competing interests

This work was supported by the Department for Environment Food and Rural Affairs, Biotechnological and Biological Sciences Research Council and Scottish Government under the Sustainable Arable LINK project (LK09136 to P.J.T). One consortium member within this LINK project is Omex Agriculture Ltd, whose Biomex Starter product contains *Bacillus amyloliquefaciens* FZB42*.*

## Authors’ contributions

PJT contributed to the design of the study, performed experimental procedures, analysed data and participated in the drafting of the manuscript. DWO contributed to the design of the study and performed experiments, and analysed data. JRH, PAW and DLJ contributed to the design of the study, analysis of data and drafting of the manuscript. All authors have read and approved the final manuscript.

## Supplementary Material

Additional file 1: Figure S1 The impact of seed dressing with *B. amyloliquefaciens* FZB42 on plant biomass, total Pi uptake or plant Pi concentration after three weeks growth. Results of a three-week pot experiment where plants that had been seed dressed at sowing with either *B. amyloliquefaciens* FZB42 or LB media as the untreated control were grown in either low Pi (A, C, E) or high Pi soil (B, D, F). A – B Total Pi acquired per plant; C – D Total dry matter yield per plant; E – F P concentration in plant tissue. For A - F n > 23 for each treatment, error bars are SEM and treatments were not significantly different from each other using Student’s t-test (p > 0.05). Click here for file
