# Supplementary figures and images for: Auxin secretion by Bacillus amyloliquefaciens FZB42 both stimulates root exudation and limits phosphorus uptake in Triticum aestivum
Source: BMC Plant Biol. 2014 Feb 21;14:51. doi: 10.1186/1471-2229-14-51 (PMC4015440; doi:10.1186/1471-2229-14-51)

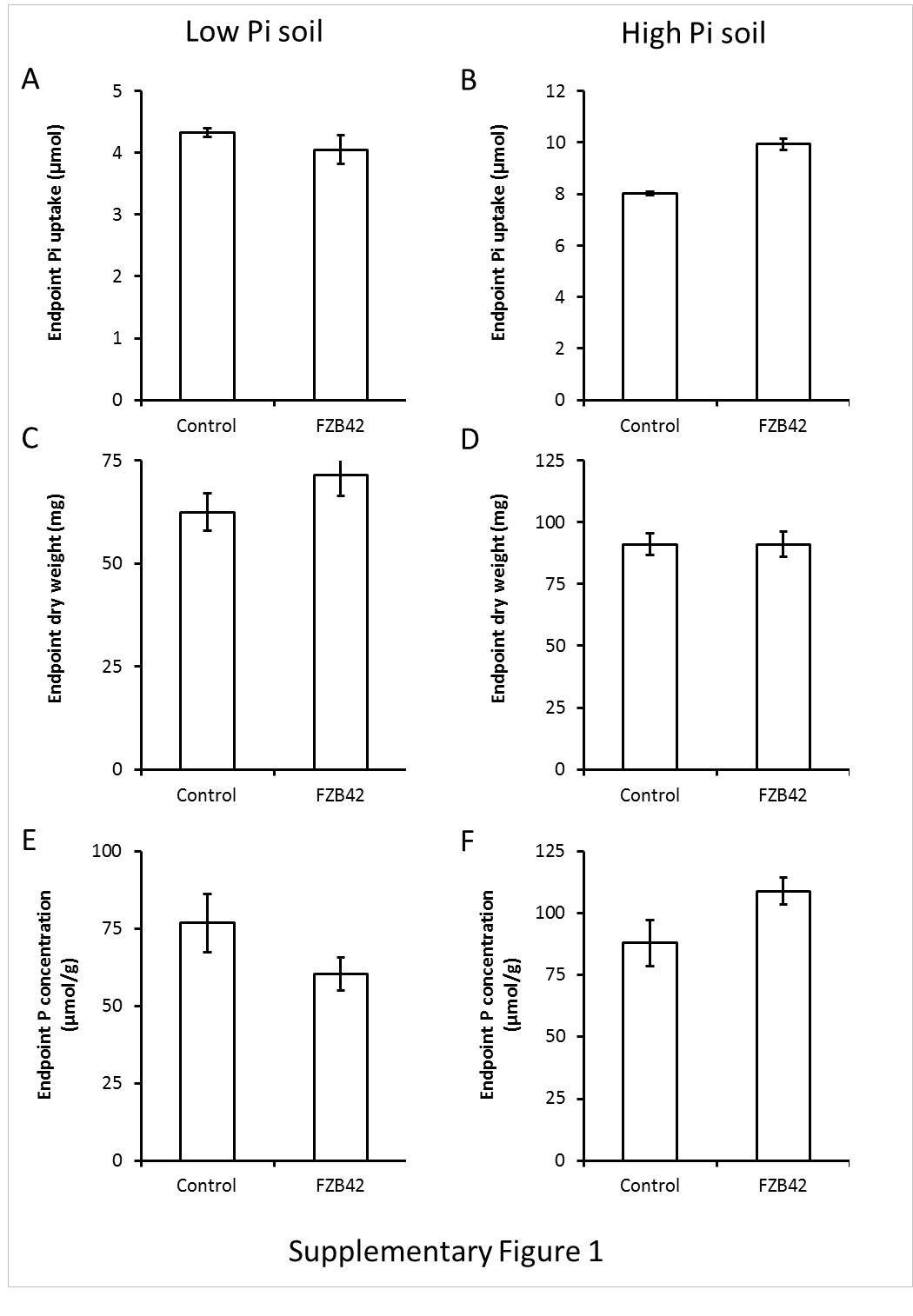

Supplement: Additional file 1: Figure S1 — The impact of seed dressing with B. amyloliquefaciens FZB42 on plant biomass, total Pi uptake or plant Pi concentration after three weeks growth. Results of a three-week pot experiment where plants that had been seed dressed at sowing with either B. amyloliquefaciens FZB42 or LB media as the untreated control were grown in either low Pi (A, C, E) or high Pi soil (B, D, F). A – B Total Pi acquired per plant; C – D Total dry matter yield per plant; E – F P concentration in plant tissue. For A - F n > 23 for each treatment, error bars are SEM and treatments were not significantly different from each other using Student’s t-test (p > 0.05). [file 1471-2229-14-51-S1.jpeg]
